# Menstruation in Old Age

**Published:** 1856-11

**Authors:** 


### MENSTRUANION IN OLD AGE.

J. J. Dixon, M. D., Ashland, Tennessee, in a business letter, gives the following interesting item:

"In a few lines, I wish to record a brief account of a singular case to which I was this day called. The patient was an old lady, aged 67, who is now menstruating; she is the mother of eight children; her menses ceased nineteen years ago, since which time she has enjoyed respectable health. Menstruation returned eleven months since, and has now occurred, in all, six times. She has not suffered any serious difficulty until the present period, and her symptoms now seem to be only those usually attendant upon painful menstruation."

[We should be glad to receive from Dr. Dixon some further account of the progress of the case described in the above communication, as we should be strongly inclined to suspect *disease* of the uterus, and not a mere resumption of its lost physiological action.—EDITORS.]

